# Rewiring Intercellular Communication with Self-Assembling Nanofibers

**DOI:** 10.1021/acsnano.6c07710

**Published:** 2026-06-23

**Authors:** Ludovico Aloisio, Vito Vurro, Alberto D. Scaccabarozzi, Fabio Marangi, Elena Feltri, Matteo Moschetta, Chiara Florindi, Nicol Spallacci, Soraia Flammini, Mattia Zangoli, Francesco Lodola, Mario Caironi, Jaime Martin, Francesca Di Maria, Guglielmo Lanzani

**Affiliations:** 1 Dipartimento di Fisica, 274268 Politecnico di Milano,Piazza L. da Vinci 32, Milan 20133, Italy; 2 Center for Nano Science and Technology, 403543 Istituto Italiano di Tecnologia,Via Rubattino 81, Milan 20134, Italy; 3 Department of Biotechnology and Biosciences, 204547 University of Milan-Bicocca, Piazza della Scienza, 2, Milan 20126, Italy; 4 Institute for Organic Synthesis and Photoreactivity (ISOF), National Research Council of Italy (CNR),Via P. Gobetti 101, Bologna 40129, Italy; 5 Universidade da Coruña, Campus Industrial de Ferrol, CITENI,Esteiro, Ferrol 15403, Spain

**Keywords:** intracellular self-assembly, nanofibers, organic semiconductors, bioelectricity, gap junctions

## Abstract

Intercellular electrical coupling mediated by gap junctions plays a central role in signal transmission in many biological systems. Its disruption contributes to cardiac and neurological disorders, as well as impaired wound healing and tumor progression. Restoring direct electrical communication between cells, however, remains challenging, particularly without genetic manipulation or the delivery of preformed devices across cellular membranes and interfaces. The small conjugated molecule DTTO (2,6-diphenyl-3,5-dimethyl-dithieno­[3,2-b:2′,3′-d]­thiophene-4,4-dioxide) self-assembles inside living cells into supramolecular nanofibers, which can extend between neighboring cells and connect their cytoplasm. Here, we show that these fibers also establish functional electrical coupling between cells: dual patch clamp recordings demonstrate restored signal transmission even when native gap junctions are pharmacologically suppressed, while control experiments show that the recovered signal transmission does not result from nonspecific membrane poration associated with fibers crossing the membrane. Electrical characterization of DTTO fiber networks shows that these structures support charge transport, while humidity-dependent measurements, impedance spectroscopy, and equivalent circuit modeling show that the observed electrical response is shaped by ionic and interfacial contributions from the surrounding environment. Collectively, this work establishes intracellular DTTO self-assembly as a nongenetic strategy to create functional bioelectrical connections in situ and restore electrical communication in diseased and engineered tissues.

## Graphical abstract

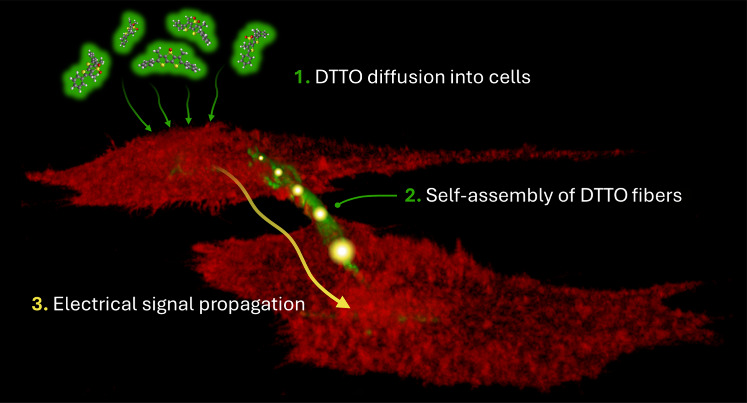

## Introduction

In biological tissues, electrical communication between cells has long been recognized as a fundamental mechanism for coordinated functions, ranging from synchronized contraction in the heart to collective repair during wound healing.^1,2^ Recently, the role of bioelectricity has gained further attention, because emerging evidence supports its function as an instructional signaling cue for fundamental cellular physiology, embryonic development and morphogenesis, regeneration, and human diseases, including cancers.^3–6^ One of the most direct and efficient mechanisms of cell-to-cell electrical communication is obtained by connexins assembling into gap junctions: membrane-spanning protein complexes that form aqueous channels between neighboring cells.^7,8^ This form of communication is particularly crucial in tissues requiring rapid, coordinated responses, such as cardiac and smooth muscle, where electrical signals propagate through gap junctions to regulate contraction.^9,10^ It also plays a major role in maintaining tissue homeostasis, enabling metabolic coupling, and mediating immune responses.^11^

Pathological conditions may disrupt the functionality of gap junctions, leading to severe consequences for tissue health. Mutations in connexin genes, oxidative stress, chronic inflammation, and ischemia are among the factors that impair gap junction formation or function.^12^ For instance, the loss of gap junction connectivity disrupts electrical conduction, increasing the risk of arrhythmias, neuronal dysfunction and synaptic loss.^13,14^ Finally, loss of gap junction functionality can also promote the progression of cancer, while functional gap junctions often suppress tumor growth.^15^

Given these roles, restoring gap junction functionality represents a compelling therapeutic strategy for multiple pathological conditions.^16^ Pharmacological agents that upregulate connexin expression or enhance gap junctional communication, such as rotigaptide, have yielded encouraging results.^17^ Other strategies aim to mitigate oxidative stress or inflammation to prevent connexin degradation and preserve junctional integrity.^18^ However, these approaches remain intrinsically dependent on the cellular machinery regulating connexin expression, trafficking, and assembly, and are limited by poor targeting, complex delivery requirements, and limited long-term stability, making the effective restoration of intercellular communication a persistent biomedical challenge.^19,20^

In this work, we demonstrate the restoration of intercellular electrical coupling using intracellularly self-assembled DTTO nanofibers. Such in situ formation from small, cell-permeable monomers can simplify delivery and help reach regions that are difficult to access with preformed architectures.^21^ DTTO is a small conjugated molecule previously shown to efficiently enter living cells and reproducibly self-assemble into ordered supramolecular nanofibers. These fibers can grow over several cell lengths and span multiple cells, making DTTO a promising candidate for mediating electrical signal transmission in living systems.^22^ We show that DTTO nanofibers can bridge neighboring HEK-293T cells and restore electrical coupling when native gap junctions are pharmacologically inhibited. A simple electrical model reproduces the main experimental observations and captures the key physical mechanisms underlying the coupling. The model indicates that the intermembrane fibers behave as weak conductors, consistent with independent electrical characterization.

## Results and Discussion

### DTTO Fiber Formation and Characterization

DTTO is a fluorescent small molecule (molecular structure shown in the inset of Figure 1) that, when added to the culture medium in solution, spontaneously permeates the plasma membrane. Due to its hydrophobic nature, once inside the cell, DTTO likely partitions into cytoplasmic lipid-rich compartments, including vesicles, leading to local cytoplasmic accumulation rather than detectable nuclear accumulation (Figure 1A).^23^ As previously reported, once internalized, DTTO molecules self-assemble into bright, one-dimensional fibers with cross-sectional areas of ∼0.1–0.3 μm^2^ and tens of microns in length (Figure 1B). These fibers grow within the cytoplasm, bending to follow the local cellular geometry. When they reach the plasma membrane in regions where another cell is present, they often cross the membrane and continue growing into the adjacent cell, while growth is halted in the extracellular environment.^24^ In a recent study, we showed that intracellular fiber formation follows a nonclassical crystallization (NCC) pathway in which DTTO first accumulates into clusters, where quasi 1D aggregates then develop.^25^ These structures have been observed in different cell lines and do not induce apparent cytotoxic effects.^26^ The fibers also appear mechanically compliant, bending with intracellular dynamics without obvious functional disruption.^25^ Grazing Incidence Wide Angle X-ray Scattering (GIWAXS) patterns of DTTO nanofibers extracted from cells, redispersed, and deposited onto silicon substrates reveal multiple isotropic diffraction maxima appearing in both the low-*q* and high-*q* regions (Figure 1E). The presence of several sharp reflections distributed over a wide-*q* range indicates that the fibers possess a high degree of structural order. The pronounced diffraction peaks at low *q* suggest the existence of long-range molecular periodicities, the innermost of which corresponds to distances ∼1.2–1.6 nm. High-*q* diffractions are associated with close π-π packing between adjacent molecules, with a stacking distance of ∼3.3 Å (diffraction patterns are reported in Figure S1). Overall, the pattern denotes a well-defined crystalline arrangement, indicating that these biogenerated fibers are structurally compatible with intermolecular long-range charge transport, as also recently demonstrated by spectroscopic characterization.^27^

**Figure 1:**
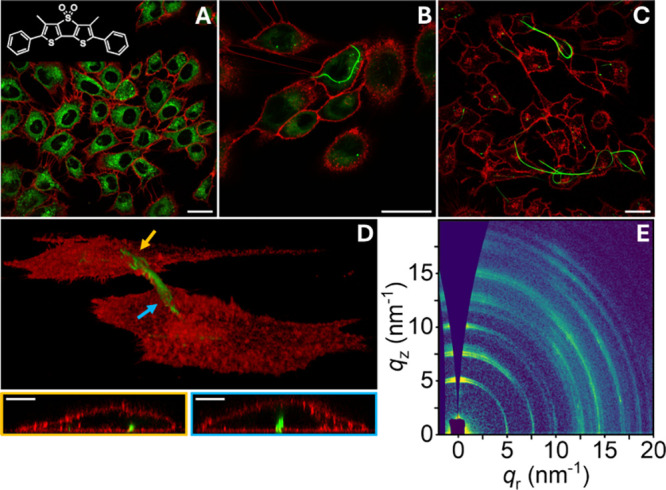
Intracellular DTTO fiber formation in living cells and GIWAXS analysis. Top left inset: molecular structure of the DTTO molecule. Laser scanning confocal microscope images depicting HEK-293T cells following DTTO exposure (green: DTTO fluorescence; red: cell membranes stained with CellMask Deep Red fluorophore): (A) immediately after exposure; (B) 3 h after exposure, showing intracellular fiber formation; (C) 12 h after exposure, showing fibers spanning between adjacent cells. Scale bars: 25 μm. (D) 3D image obtained by Z stack acquisition of two HEK-293T cells connected by a DTTO fiber. Below, orthogonal XZ sections extracted from the same data set at the positions indicated by the colored arrows in the main image. The color of each frame matches the corresponding arrow, identifying the position from which each section was taken. Scale bars: 5 μm. (E) 2D-GIWAXS patterns of extracted DTTO fibers.

### Electrical Intercellular Coupling

Nanofibers can pierce the plasma membrane of adjacent cells^25^ thereby connecting their cytoplasm. To test whether this enables intercellular communication, we carried out double patch-clamp recordings on neighboring HEK-293T cells with or without intercellular fiber coupling.^28,29^ In these experiments one pipette applied a current stimulus (500 pA, 20 ms) to cell A and we recorded the signal in current or voltage clamp configuration from cell B, as schematically represented in Figure 2A. Although HEK-293T cells are often considered gap junction-deficient,^30^ there is evidence that they can occasionally form functional gap junctions.^31^ The presence of connexin 43 (Cx43) in HEK-293T cells was verified by immunostaining and provides a valuable model system for our experiment. Indeed, in adjacent cells, current injection in one cell could lead to propagation of membrane depolarization to the neighboring cell (see Figure S2). Immunostaining of DTTO-treated cells did not reveal evidence of enhanced endogenous gap junction formation, as assessed by Cx43 distribution and abundance (Figure S3). To suppress this endogenous communication and establish a clear experimental baseline, we used the gap junction blocker carbenoxolone (CBX).^32,33^

**Figure 2:**
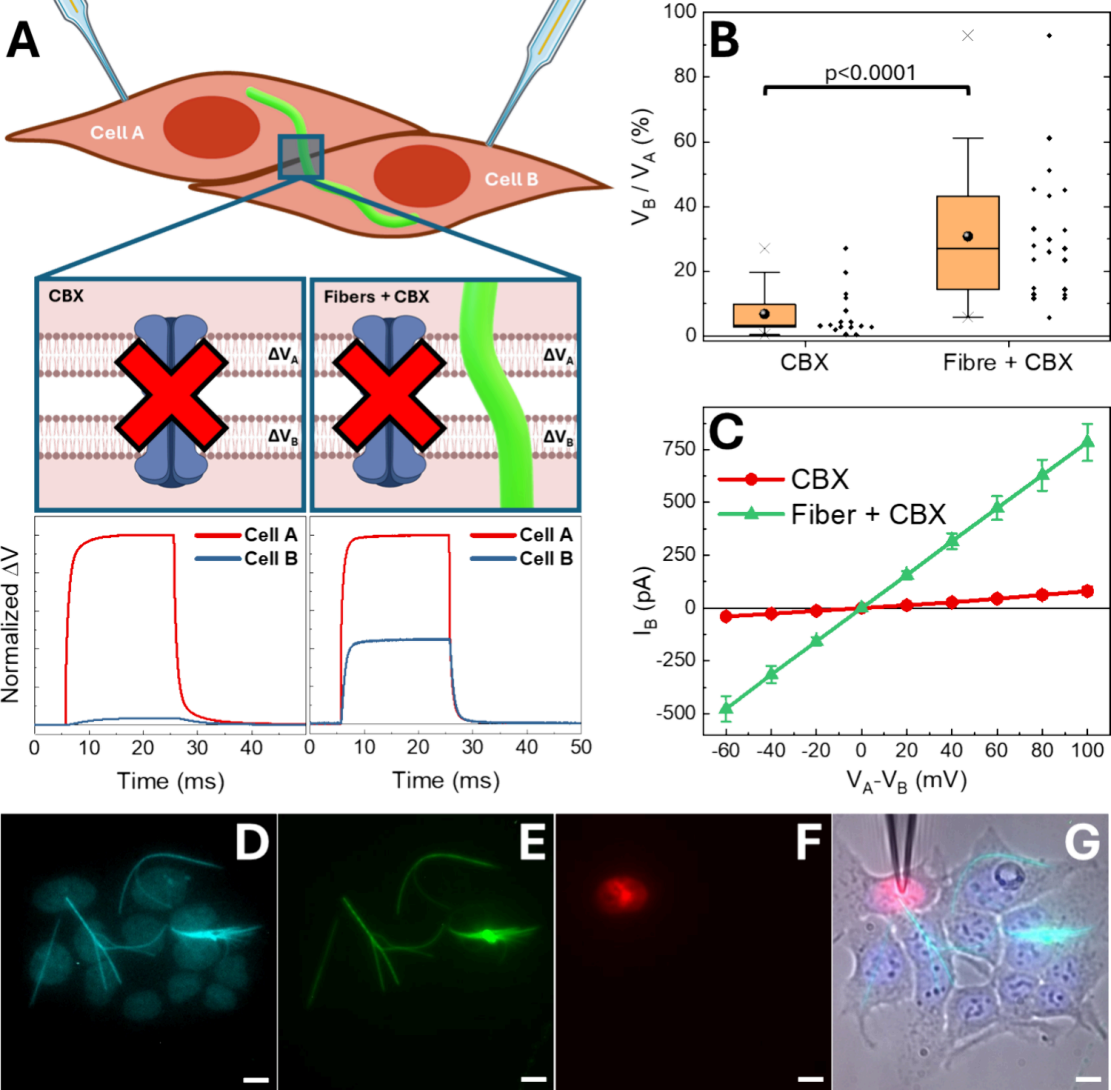
Electrical intercellular coupling through DTTO fibers. (A) Schematic of the experiment and representative membrane potential traces recorded in cell A and cell B during a current pulse injected in cell A. Traces are normalized from 0 (resting potential) to 1 (maximal depolarization in cell A). (B) Box plot of coupling ratios for CBX (*N* = 16) and fiber + CBX (*N* = 21). Boxes show the interquartile range with median; whiskers extend to 1.5 × IQR; points indicate individual measurements. P values were computed using a two-tailed Mann–Whitney U test (*p* < 0.0001). (C) *I*–*V* curves: cells were held at −20 mV and voltage steps from −80 to +80 mV (20 mV increments) were applied to cell A while current was recorded in cell B; the *x*-axis reports the intercell voltage difference. (D–G) Fluorescence microscopy images (40X) of HEK-293T cells connected by DTTO fibers during whole cell patch clamp. The patch pipette contained propidium iodide to probe intercellular pores. Channels show (D) Hoechst plus DTTO fibers, (E) DTTO fibers, (F) propidium iodide, and (G) composite image, superimposed with brightfield. Scale bars: 10 μm.

HEK-293T cells were treated with DTTO 24 h before the electrophysiology experiments to allow intercellular fiber formation. Immediately before recordings, samples were incubated with CBX under standard cell culture conditions for 30 min. To ensure the validity and reproducibility of the results, only cells with a clearly visible separating membrane were selected (Figure S4). Experimental results are reported in Figure 2. As expected, CBX almost completely suppressed electrical communication, leaving a residual membrane depolarization of less than 7% in the second cell upon current injection into the first cell. Remarkably, in the presence of pharmacologically suppressed gap junctions, intercellular electrical communication is restored when adjacent cells are connected by DTTO fibers. The “transmitted” potential change in cell B is in the order of 30% of that in directly stimulated cell A (Figure 2B). This validates DTTO fibers as artificial intercellular conductive connectors, capable of restoring electrical coupling between cells.

Additionally, we recorded intercellular *I*-*V* characteristics, measuring the current response of cell B upon membrane polarization of cell A (Figure 2C). Cell B was measured in voltage clamp at *V*_*hold*_ = – 20 mV while a standard voltage-step protocol (−80 mV to +80 mV, in 20 mV increments) was applied to cell A. The current response of cell B reflects the degree of intercellular coupling, further demonstrating that the presence of bridging fibers enables efficient electrical communication.

To assess the mechanism by which DTTO fibers act as artificial gap junctions, we examined whether signal transmission could arise from ion transport through small membrane disruptions caused by fibers piercing. As an initial indication, we analyzed a configuration in which two cells that were not in close contact were connected by a DTTO fiber spanning the gap between them (Figure S5). These cells still exhibited signal transmission of approximately 26%, supporting the role of the fiber itself as an electrical bridge. This geometry makes it unlikely that a thin cytoplasmic extension enclosed by membrane, if present, could fully account for electrical signal propagation over this distance. We then directly tested whether fiber crossing was associated with membrane permeabilization by loading patch clamp pipettes with propidium iodide (PI), a fluorescent dye commonly used to detect compromised membrane integrity.^34^ PI was loaded into patch-clamp pipettes and delivered directly into patched cell A, where it was allowed to diffuse through the intracellular solution. Once PI had diffused throughout cell A, we applied the same current steps used in the double patch-clamp measurements. Fluorescence imaging performed 1 h later showed that PI remained confined to patched cell A and did not appear in the adjacent cell B (Figure 2D–G and Figure S6). These observations indicate that DTTO fibers do not form membrane pores or compromise membrane integrity.

### Electrical Cell Coupling Model

We derived an equivalent circuit for the double patch clamp experiment described in the previous section, in which the electrical coupling between the two cells is represented by RC elements, as illustrated in Figure 3A. The system of equations describing the time evolution of the intracellular voltages, *V*_A_(*t*) and *V*_B_(*t*), under current injection into cell A was solved numerically; details are provided in the Supporting Information. The resulting membrane depolarization in both cells closely reproduced the experimental voltage recordings (Figure 3B,C) when all parameters were fixed at typical physiological values and the junction resistance was optimized (Table S1). A value of *R*_*j*_ ≈ 10^2^ MΩ reproduced the traces measured for coupled cells, whereas a junction resistance about 1 order of magnitude larger was required to reproduce the CBX condition.

**Figure 3:**
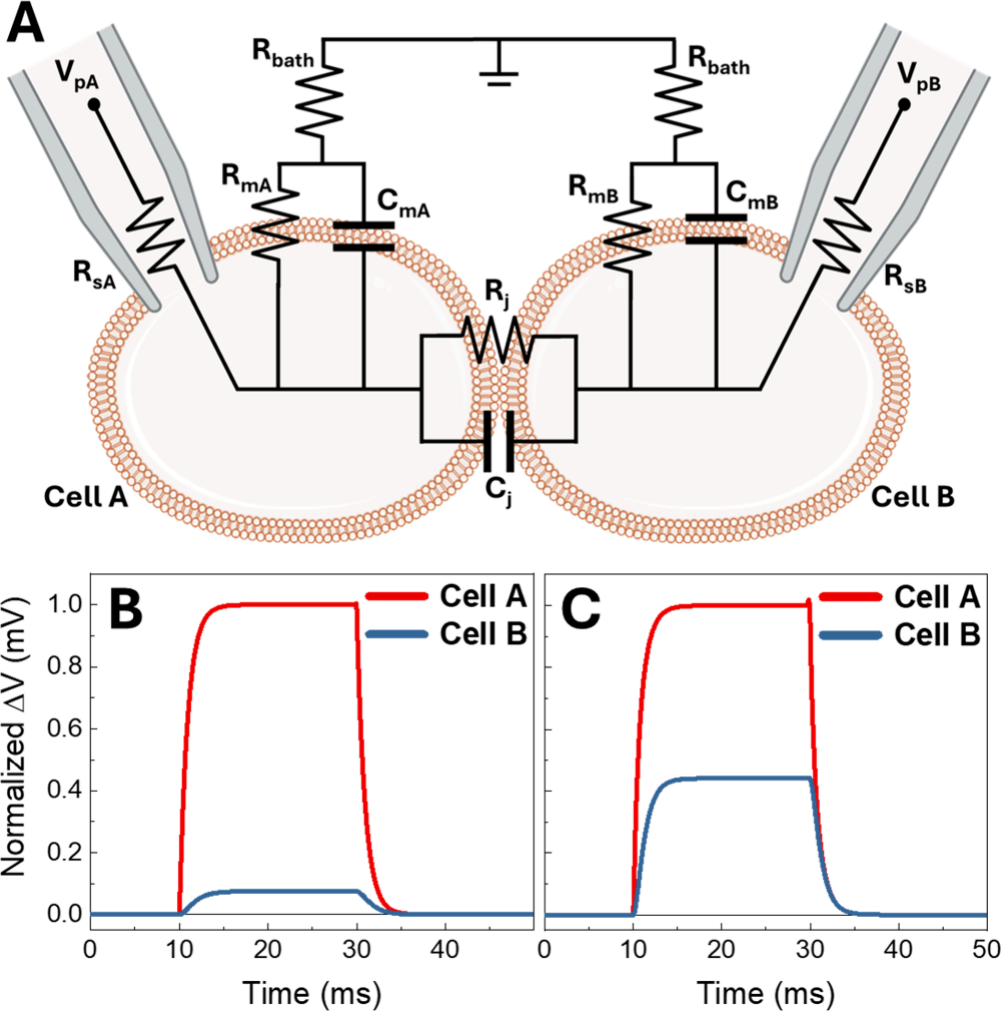
Equivalent circuit model and simulated membrane responses. (A) Equivalent circuit for the double patch clamp configuration in HEK-293T cells. *V*_p_, pipette voltage; *R*_s_, series access resistance; *R*_m_, membrane resistance; *C*_m_, membrane capacitance; *R*_bath_, extracellular solution resistance to the counter electrode; *R*_j_, junctional resistance; *C*_j_, junctional capacitance. Subscripts A and B denote the two cells. (B, C) Membrane potential traces simulated from the equivalent circuit model for cells A and B during a 500 pA, 20 ms current pulse injected into cell A for (B) CBX and (C) fiber + CBX. Traces are normalized from 0 (resting potential) to 1 (maximal depolarization in cell A).

We next asked which transport properties of DTTO underlie the electrical behavior observed in the fibrous state. We first examined spin-cast DTTO thin films in conventional transistor geometries under dry inert nitrogen atmosphere. These films exhibited reproducible p-type operation (see Figure S7), with a field-effect hole mobility on the order of μ ≈ 10^–6^ cm^2^/(V s), in agreement with literature on related thiophene-based systems.^35,36^ These results show that DTTO in the solid state can transport electronic charge, but they do not provide information on the performance of the fibrous form due to differences in crystal packing. As shown above, DTTO fibers exhibit a high degree of crystallinity (Figure S8A,B), and charge transport in molecular semiconductors is highly sensitive to crystal packing and microstructure.^37^ Considering the difficulty of extracting and cleaning fibers from cells, we prepared one-dimensional DTTO aggregates by drop-casting from DMSO as a model for the intracellular fibers. GIWAXS measurements confirmed their structural similarity to the intracellularly formed fibers (Figure S8C,D).

As reported in Figure 4A, these one-dimensional aggregates likewise supported charge transport, with an apparent hole mobility of μ ≈ 10^–5^ cm^2^/(V s) (Figure S9), exceeding that of the spin-cast films and consistent with their greater structural order. This value likely underestimates the intrinsic mobility along individual fibers, because the devices contain randomly distributed aggregates and the analysis assumes that all of them contribute equally to transport, while grain boundaries between adjacent structures may further limit electrical connectivity (Figure S10).^38^

**Figure 4:**
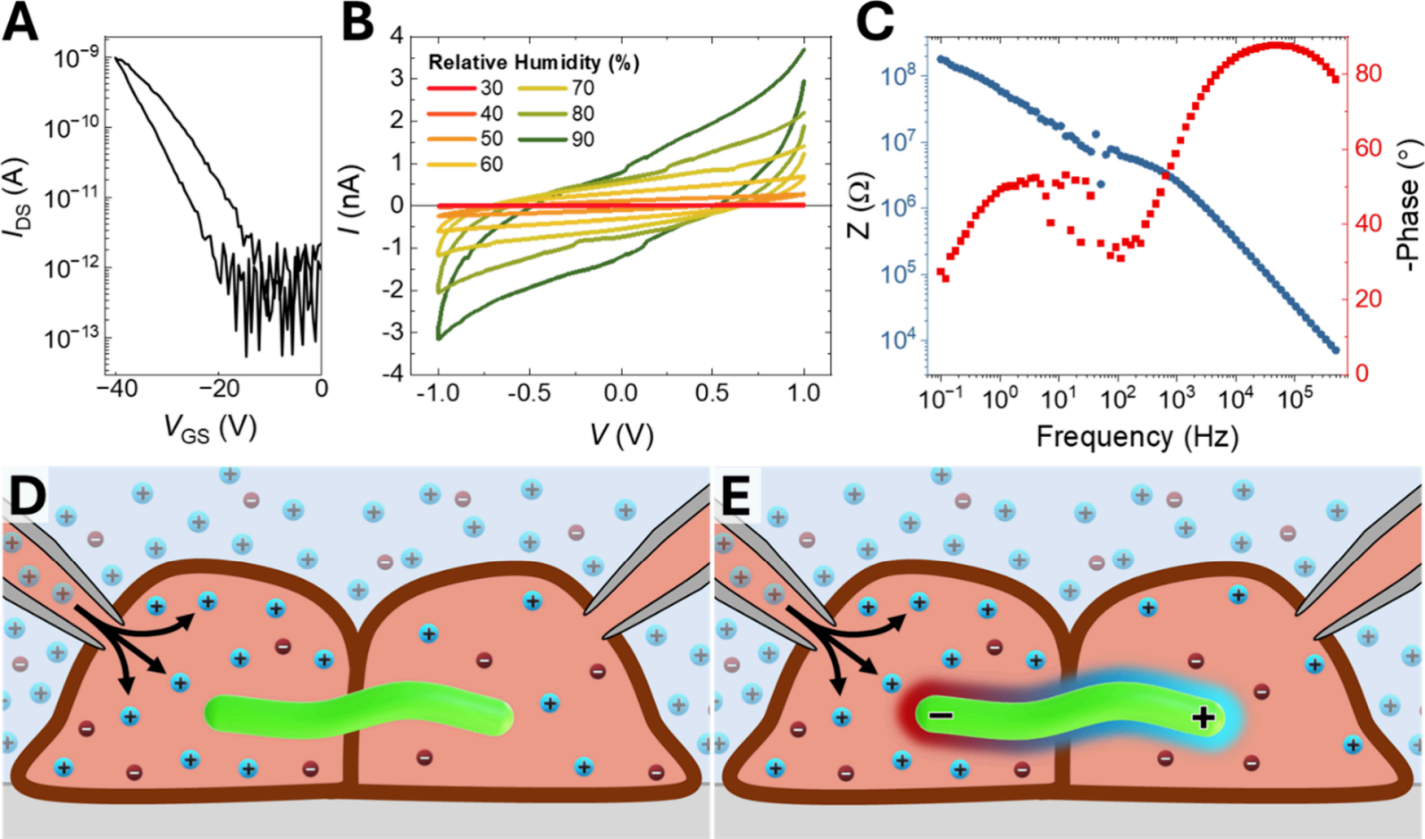
Electrical characterization of DTTO fibers and signal transduction mechanism. (A) Representative transfer characteristic curves of bottom gate transistors based on DTTO aggregates grown by DMSO solution evaporation, measured under nitrogen glovebox conditions. (B) *I*–*V* characteristics of the same DTTO aggregates sample measured at increasing relative humidity. (C) Electrochemical impedance spectroscopy of the same DTTO aggregates sample at 90% relative humidity, shown as a Bode plot of impedance modulus (blue) and the phase (red). (D, E) Cartoon representing the electrical coupling mechanism through DTTO fibers: (D) A positive current injected into cell A increases the intracellular positive charge density. (E) The resulting potential difference drives a DTTO fiber-mediated response that redistributes positive ions in cell B toward the plasma membrane, leading to depolarization.

Although these measurements establish that DTTO supports electronic charge transport, this evidence cannot fully account for the electrical behavior observed in cells under physiological conditions, where external gating is not present. This points to an additional role of the surrounding environment, as further discussed in the Supporting Information. We therefore examined the electrical response of DTTO fibers under controlled relative humidity (RH) using current-voltage measurements in a lateral two-terminal coplanar geometry. As shown in Figure 4B, below 50% RH, the current remained negligible. Above this threshold, a measurable current became evident and increased progressively with humidity, while the bare substrate at 90% RH showed negligible current, ruling out humidity-induced substrate leakage (Figure S11). The current-voltage characteristics also became nonlinear and displayed pronounced hysteresis between forward and reverse sweeps, indicating the emergence of a distinct humidity dependent electrical response. In addition, under humid conditions, the current measured at constant applied bias (−1 V) did not remain constant but decayed over time (Figure S12).^39^ Together, these observations suggest that the electrical response of hydrated DTTO fibers cannot be described as simple electronic transport alone and motivate a more detailed analysis of its origin.

We further probed the nature of this humidity dependent response by impedance spectroscopy on DTTO fibers under controlled RH in the same coplanar geometry (Figure 4C). At low relative humidity, the impedance spectra were indistinguishable from the device background, consistent with the negligible current observed in the current–voltage measurements. Above 50 to 60% RH, an additional response was observed (Figure S13), with a nonideal and dispersive character that was well described by two characteristic relaxation processes (further discussion in the Supporting Information, Figure S14A). Unlike H_2_O alone (Figure S14B), the DTTO samples did not show the same predominantly ionic blocking response. Instead, the phase remained around 50° over a broad intermediate frequency range, consistent with a distributed and disordered electrical response rather than an ideal capacitive one.^40^ A sample containing the same amount of H_2_O together with DTTO fibers also remained distinct from H_2_O alone, further showing that the measured behavior does not arise simply from an ionic leakage path but is modulated by the presence of the DTTO fiber network (Figure S14C). Consistent fits across these samples further support this interpretation, indicating an additional dispersive component associated with the fiber network when present (Table S2). Overall, humidity strongly alters the electrical behavior of DTTO fibers through humidity-induced interfacial polarization and ionic effects, rather than clear bulk ionic conduction through DTTO itself. These observations are likely relevant to the biological setting, where DTTO fibers are naturally surrounded by aqueous media and biomolecules.

Consistent with these observations, Figure 4D,E illustrates the proposed working model for signal transduction. Injection of a positive current step, *I*_*inj*_, into cell A changes its membrane potential by *ΔV* = *R*_*m*_ *×I*_*inj*_, where *R*_*m*_ is the total membrane resistance. In the presence of a DTTO fiber bridging cell A and cell B, this perturbation creates an electrochemical imbalance between the two cells that polarizes the fiber. The resulting polarized state can drive local ionic reorganization at the fiber-cell interfaces and within the surrounding intracellular environment, thereby leading to depolarization of cell B.

## Conclusions

In this work, we report that DTTO fibers crossing adjacent cell membranes act as artificial gap junction, establishing functional intercellular electrical communication. This was demonstrated by dual patch-clamp recordings in cells where natural communication was pharmacologically suppressed. Importantly, this functional recovery occurs without evidence of membrane poration, capacitive artifacts or any effect on cell viability. We attribute this restored electrical coupling to the electrical properties of the DTTO fiber nanoarchitecture. Our measurements show that DTTO fibers support electronic charge transport, shaped by the surrounding environment through interfacial polarization and ionic effects. This is particularly relevant in cells, where the characteristic length scales are far shorter than in transistor devices and the intracellular environment is highly hydrated and rich in mobile ions.

Our findings highlight the potential of supramolecular self-assembly of exogenous small molecules to construct intracellular “nano-architectures”. It is an example of the emerging concept of self-assembled functional structures: the idea of simple injection of monomers or units into a living cell-tissue-organism, that by auto-organization create functional structures.^41,42^ While this is naturally occurring in cells (e.g., cytoskeletal dynamics, organelle formation), here we refer to the intentional design or harnessing of such processes for therapeutic or technological applications.^43^ The unique ability of DTTO nanofibers to physically and electrically bridge adjacent cells offers a novel artificial strategy to mitigate the consequences of gap-junction dysfunction. Gap-junction alterations have been implicated in a wide range of disorders, including congenital sensorineural hearing loss (a leading cause of hereditary deafness), cardiac arrhythmias that may progress to heart failure, Charcot-Marie-Tooth disease (an inherited peripheral neuropathy), and certain forms of congenital cataract.^44,45^ For example, in cardiac tissue, this approach could potentially help restore signal propagation across poorly coupled regions where fibrosis or gap-junction remodeling disrupts action potential conduction. In peripheral neuropathies, artificial conductive bridges could also provide a future strategy to support electrical signal transmission across regions where cellular communication or tissue conductivity is impaired. Although our findings highlight the potential of DTTO nanofibers as a platform to restore intercellular electrical coupling, clinical translation will require extensive additional work, including precise spatial control over fiber formation and orientation, long-term safety assessment, and validation in physiologically relevant *in vitro, ex vivo* and *in vivo* models.

## Materials and Methods

### Cell Culture Maintenance

In vitro experiments were conducted using HEK-293T (human embryonic kidney) cells obtained from ATCC. The cells were cultured in T-25 flasks containing Dulbecco’s modified Eagle medium high glucose (DMEM-HG), supplemented with 10% heat-inactivated fetal bovine serum (FBS) and 1% GlutaMAX (0.5 mm, Invitrogen). The culture flasks were kept in a humidified incubator at 37 °C with 5% CO_2_.

Once confluent, cells were enzymatically detached using a 1x trypsin-EDTA solution, seeded onto sterilized substrates, and allowed to grow for at least 24 h before experiments. Prior to cell seeding, a fibronectin layer (2 μg/mL in PBS buffer) was applied to the sample surface and incubated at 37 °C for at least 1 h to enhance cell adhesion.

### DTTO Treatment

HEK-293T cells were plated at a density of approximately 12,000 cells cm^–2^ in a 12-well tissue culture plate, with 1 mL of complete culture medium per well. DTTO was first dissolved in DMSO at a concentration of 2.5 mg/mL to prepare a stock solution, then diluted in serum-free DMEM to a final concentration of 25 μg/mL before being added to the cells. The cells were incubated at 37 °C with 5% CO_2_ and 95% relative humidity for 1 h. Following incubation, any remaining dye in the solution, along with DTTO aggregates that did not penetrate the cell membrane, was removed by washing with PBS and adding fresh medium.

### Confocal Imaging

Confocal imaging was performed using a Nikon Eclipse Ti2 inverted microscope equipped with a Nikon A1 confocal scanning unit. Image acquisition was carried out using NIS-Elements, Nikon Imaging Software. Cells were seeded at a density of 12,000 cells cm^–2^ and incubated for 24 h before the experiment. Cells were then treated with DTTO for 1 h as described in previous paragraph. After incubation, samples were washed with PBS to remove unbound molecules.

Right before imaging, samples were incubated for 5 min with CellMask Deep Red (Thermo Fisher) to stain the plasma membrane. After washing with PBS, confocal imaging was performed using a 60× objective. DTTO and CellMask were excited using lasers at 403.3 nm (detection channel: 500–550 nm) and 640 nm (collection channel: 663–743 nm), respectively. Image analysis was conducted using Fiji (ImageJ).

### DTTO Fiber Harvesting

DTTO fibers were purified from whole-cell lysates using a lysis buffer containing 50 mM Tris-HCl (pH 7.4), 1% Triton X-100, 5 mM EDTA, 150 mM NaCl, 1 mM Na_3_VO_4_, 1 mM NaF, 1 mM PMSF, 10 μM benzamidine-HCl, and protease inhibitors (10 μg/mL aprotinin, 10 μg/mL leupeptin, and 10 μg/mL pepstatin A). The resulting solution was washed multiple times with fresh lysis buffer by centrifugation (1550 rpm, 20 min, 4 °C) and stored at – 80 °C for future use.

### DTTO Aggregates Production from DMSO Solution

Fiber-mimicking DTTO aggregates were prepared without cells by slow evaporation of DMSO. DTTO was diluted into DMSO at 1 mg/mL, 100 μL of solution was drop-cast on a glass substrate and let evaporate in vacuum at room temperature for 1 week. Before using them, all substrates were cleaned by sonication in acetone and isopropanol, followed by UV–O_3_ treatment at 100 W for 5 min. Samples were then inspected through confocal microscopy. For GIWAXS, the same preparation was carried out, casting the solution on silicon substrates.

### Grazing-incidence wide-angle X-ray scattering

GIWAXS (Grazing Incidence Wide Angle X-ray Scattering) measurements were carried out at the BL11-NCD-Sweet beamline, located at the ALBA Synchrotron Radiation Facility in Barcelona, Spain. For these measurements, a Rayonix WAXS LX255-HS detector, with a high resolution of 1920 × 5760 pixels, was employed to collect the scattering signals, ensuring high precision in data acquisition. The incident beam energy was set at 12.4 keV, and the sample-to-detector distance was carefully calibrated to 201.655 mm to optimize the scattering pattern collection. The angle of incidence (αi) was maintained at a low value of 0.12°, a standard parameter to minimize penetration depth while maximizing surface scattering. Each exposure had a set duration of 5 s to capture the necessary diffraction data.

The 2D GIWAXS patterns obtained were then corrected with a MATLAB script, accounting for the scattering vector components to ensure accurate representation of the crystalline structures. Thin films of various DTTO samples, including DCM, fibers and DMSO-derived aggregates, were prepared by casting onto highly doped silicon substrates. This casting process followed the same procedures as those used for the fabrication of samples used for other experiments. Fibers were deposited on the silicon substrates and allowed to dry overnight, ensuring that the samples were fully stable before measurements were conducted.

### Cx43 Immunostaining

HEK cells were plated and treated with DTTO as previously described. After 2 days of culturing, samples were fixed with Antigenfix solution (Diapath) and rinsed with PBS 3 times. Triton X-100 0.1% (Sigma-Aldrich) was used for permeabilization. Blocking was performed using 5% bovine serum albumin (BSA; Sigma–Aldrich) in PBS for 1 h at room temperature. Samples were then incubated overnight at room temperature with rabbit anti-connexin43 primary antibody (ABCAM) at a 1:300 dilution in 1% BSA/PBS. Following two washes in PBS, samples were incubated for 2 h at room temperature with Alexa Fluor 647 donkey antirabbit IgG (H+L) secondary antibody (Thermo Fisher Scientific) at 1:500 in 1% BSA/PBS. After three PBS washes, nuclei were counterstained with Hoechst 33342 (1 μg mL^–1^ in PBS) for 10 min at room temperature in the dark, followed by two PBS washes. Samples were stored in PBS at 4◦C until imaging.

### Patch Clamp Measurements

Standard patch-clamp recordings were performed using an Axopatch 700B amplifier (Axon Instruments, San Jose, CA, USA), integrated with a Nikon Eclipse Ti inverted microscope to facilitate high-quality visualization of the cells during the measurements. HEK-293T cells were analyzed in the whole-cell configuration using freshly pulled glass pipettes (4–7 MΩ) filled with a carefully prepared intracellular solution, which included (in mM): 12 KCl, 125 K-Gluconate, 1 MgCl_2_, 0.1 CaCl_2_, 10 EGTA, 10 HEPES, and 10 ATP-Na_2_. The extracellular solution, which was used to bathe the cells during recordings, consisted of (in mM): 135 NaCl, 5.4 KCl, 5 HEPES, 10 Glucose, 1.8 CaCl_2_, and 1 MgCl_2_. The acquisition of the data was managed with pClamp-11 software (Axon Instruments, San Jose, CA, USA), which enabled real-time monitoring and precise control of experimental parameters.

To ensure the accuracy and integrity of the signal, membrane currents were low-pass filtered at 2 kHz to reduce noise, and the data was digitized at a sampling rate of 10 kHz using a Digidata 1550B interface (Molecular Devices, San Jose, CA, USA). Current-clamp recordings were carefully selected based on the access resistance of the cell, with only those exhibiting an access resistance lower than 8 MΩ included in the analysis. Furthermore, both capacitance and resistance compensations were applied during the experiment to account for potential distortions in the current response due to electrode and cell membrane characteristics.

For double patch-clamp measurements, both electrodes were connected to the same setup to allow for simultaneous recording from two cells, with both cells recorded in the whole-cell configuration. This ensured that the voltage or current was applied identically to both cells, allowing for a direct comparison of their responses. The protocol for this dual configuration involved applying a specific voltage or current to one of the cells while simultaneously monitoring the signal from both cells to ensure the proper sealing of electrodes and reproducibility of the applied stimuli. The robustness of this configuration was confirmed by observing stable recordings from both cells, ensuring that both cells remained well-sealed throughout the experiment, thus allowing for reliable data collection and analysis.

To ensure the validity and repeatability of results, only cell pairs clearly showing a membrane between them were selected. To further exclude fused cells, where gap junctions or fibers would not play a relevant role in signal transduction, the membrane test mode was monitored while breaking into the cells. In a dual patch-clamp configuration, when both electrodes establish whole-cell access, applying a square wave to one electrode results in the appearance of an identical but opposite square wave on the other electrode if the two cells are fused. This occurs because the injected current spreads through the shared cytoplasm, causing a mirrored capacitive response. Conversely, if the cells are not fused, the signal remains isolated to the patched cell, confirming the presence of a separating membrane.

### Gap Junction Blocking

Three different experimental conditions were investigated using HEK-293T cells: untreated cells, cells with hindered communication (induced by exposure to CBX), and cells connected by DTTO fibers while being exposed to CBX. In the case of untreated cells, the experimental group consisted of neighboring HEK-293T cells, serving as the baseline for comparison. Non communicating cells were treated with 100 μM carbenoxolone (CBX) for 30 min to block gap junction communication between the cells. After the incubation period, these cells were analyzed while ensuring that the CBX concentration remained constant throughout the experiment to maintain the disruption of intercellular communication. Both control samples were exposed to 1% DMSO the day before experiment, reproducing the same protocol applied for DTTO treatment but in absence of the molecule itself.

For the third condition, the cells were exposed to CBX as described above, while being connected by DTTO fibers. In this case, only cells that were physically connected by the same DTTO fiber were selected for measurement, allowing for the examination of potential changes in communication due to the presence of the fiber network. Importantly, in all experimental conditions, only neighboring cells that clearly displayed a discernible membrane separating them were selected for analysis, ensuring that the observed effects were representative of intercellular interactions and not due to artifacts such as cells being in contact or overly adjacent.

### Propidium Iodide Diffusion through Junctions

To assess whether DTTO fibers induce membrane poration, propidium iodide (PI) was delivered intracellularly through the patch pipette and its distribution was monitored by fluorescence microscopy. Patch pipettes were filled with the intracellular solution supplemented with PI (50 μg/mL final concentration). A single cell (cell A) connected to neighboring cells via DTTO fibers was patched in whole cell configuration, allowing PI to diffuse into the cytosol from the pipette. After obtaining whole cell access, PI was allowed to diffuse for 10 min. Current steps (20 pulses, 500 pA, 20 ms) were then applied to the patched cell. After 1 h, fluorescence images were acquired to determine whether PI remained confined to the patched cell or appeared in adjacent cells.

Fluorescence imaging was performed on the same inverted microscope used for electrophysiology (Nikon Eclipse Ti inverted microscope) using a 40X objective, acquiring both brightfield and epifluorescence images, while the patched cell was maintained in whole cell configuration. Hoechst was added to the bath to label cell nuclei and facilitate identification of all cells in the field of view. Illumination was provided by an LED light engine (Lumencor SPECTRA III) coupled with the microscope, and fluorescence was collected using standard epifluorescence filter sets. Hoechst was excited using the 394 nm LED line and detected using a DAPI filter set (excitation 350/50 nm; dichroic 400 nm long pass; emission 460/50 nm). DTTO fluorescence was excited using the 474 nm LED line and detected using a FITC filter set (excitation 470/40 nm; dichroic 495 nm long pass; emission 525/50 nm). Propidium iodide fluorescence was excited using the 545 nm LED line and detected using a TRITC filter set (excitation 540/25 nm; dichroic 565 nm long pass; emission 605/55 nm).

### MATLAB Model on Double Patch Clamp Experiments

An equivalent electrical circuit model was developed to describe a dual patch clamp configuration on two electrically coupled cells, in which current injection was applied to one cell while membrane potentials were recorded from both. The model incorporated the main elements required to reproduce the experimental response, including passive membrane properties, intercellular electrical coupling, and the access and series resistances associated with the patch pipettes. The model was implemented in MATLAB as a numerical simulation to generate expected voltage traces for defined stimulation protocols and to enable direct comparison with experimental recordings, with circuit parameters adjusted when necessary to maximize agreement between simulations and data. A detailed description of the model and its numerical implementation is provided in the Supporting Information.

### Electrical Measurements

Conductivity measurements were performed employing a two-contact configuration based on interdigitated electrodes, fabricated using standard photolithography. A positive photoresist (AZ5214E) was spin-coated onto cleaned glass substrates at 6000 rpm for 60 s, followed by soft baking at 110 °C for 90 s. Electrode patterns were defined by exposure to 365 nm UV light using a maskless aligner (Heidelberg MLA100). The resist was subsequently developed in a metal ion-free developer (MIF726) for 25 s. Channel width was 30 mm, while different channel lengths were fabricated ranging from 2.5, 5, 10, 20, and 40 μm. To promote adhesion to the glass substrate, a thin layer of chromium (3 nm) was deposited before the gold one (30 nm) via thermal evaporation, and the two layers were deposited one on top of the other without breaking vacuum. Lift-off was performed by immersion in Technistrip 2.

Organic field effect transistors based on DTTO were fabricated in two device architectures: a) the same interdigitated Au electrodes used for conductivity measurements were used for source and drain electrodes in a top-gate bottom-contact configuration. b) Commercially available testbeds from Fraunhofer Institute, based on doped silicon substrates with thermally grown SiO_2_ acting as a dielectric (90 nm) and lithography-defined source drain electrodes (Gold, with ITO as adhesion layer) were used for both bottom-gate bottom-contact configuration (Si acts as gate electrode) and for top-gate configuration. In both device architectures, DTTO was dissolved in dichloromethane at 5 mg mL^–1^ and deposited by spin coating at 1000 rpm for 60 s, followed by annealing at 100 °C. For the top gate bottom contact devices, a Teflon dielectric layer of approximately 600 nm was subsequently spin coated on top of the DTTO film and an Al gate electrode was deposited by thermal evaporation. Electrical characterization was performed using an Agilent B1500A Semiconductor Parameter Analyzer with a probe station in a nitrogen filled glovebox or in ambient air, depending on the experiment. Humidity-controlled measurements were performed using an environmental chamber (Memmert HPP110ecoplus) and standard wiring were used to contact the sample through a cable window in the environmental chamber. To improve the contact stability, contact pads were specifically fabricated. The former consisted of cm-wide, 100 nm gold contacts, thermally evaporated by shadow mask, while the whole setup was systematically assessed to minimize the presence of parasitic currents. Thereby, a voltage sweep within 1 V range was selected, to minimize potential electrochemical reactions at high humidity levels. For the quantification of the calibration curve (Figure S11) the sample was left 1 h at 25 °C and 90% RH. To produce a reproducible humidity stimulus, the sample was left for 15 min at the given humidity level and both measured 1) at ambient conditions (30% RH) first and then upon increasing the humidity up to 90% RH, and 2) upon performing the reverse measurement from 90% RH to 30% RH. No difference was observed between the rising and decreasing humidity experiment.

### Electrical Impedance Spectroscopy

Electrical impedance spectroscopy measurements were carried out by using a potentiostat/galvanostat (Autolab, PGSTAT 302N) employing the same architecture and setup used for the other electrical measurements. The measurements were performed at the equilibrium obtained by applying a potential equal to V_off_ of the measure. The amplitude of the sinusoidal signal (V_eff_) and the integration time were set to 50 mV and 200 ms, respectively.

## Supplementary Material



## Data Availability

Data, code, and materials availability: patch clamp recordings, raw electrical characterization files, GIWAXS data, and the analysis scripts and code used for the electrical model are publicly available in Zenodo (DOI: 10.5281/zenodo.18328157).

## Notes

Supplementary text on equivalent circuit resolution (MATLAB) and electrical characterization; Figures S1–S14; and Tables S1 and S2 (PDF)

## †

Laboratory of Organic Electronics, Department of Science and Technology, Linköping University, SE-60174 Norrköping, Sweden (L.A.)

## Abbreviations

DTTO: 2,6-diphenyl-3,5-dimethyl-dithieno­[3,2-b:2′,3′-d]­thiophene-4,4-dioxide
CBX: carbenoxolone
HEK: human embryonic kidney
Cx43: connexin 43
NCC: nonclassical crystallization
GIWAXS: grazing incidence wide-angle X-ray scattering
PI: propidium iodide
RH: relative humidity
EIS: electrochemical impedance spectroscopy
DMSO: dimethyl sulfoxide
DMEM: Dulbecco’s modified Eagle medium
PBS: phosphate-buffered saline
FBS: fetal bovine serum
BSA: bovine serum albumin
EDTA: ethylenediaminetetraacetic acid
