# Supplementary material for: Rewiring Intercellular Communication with Self-Assembling Nanofibers
Source: ACS Nano. 2026 Jun 23;20(26):19116–25. doi: 10.1021/acsnano.6c07710 (PMC13348161; doi:10.1021/acsnano.6c07710)
Supplement: Supplementary file 1 [file nn6c07710_si_001.pdf]

Supplementary Information for

Rewiring intercellular communication with self-assembling nanofibers

Ludovico Aloisio[1,2]†, Vito Vurro[2], Alberto D. Scaccabarozzi[1,2], Fabio Marangi[2], Elena Feltri[2], Matteo Moschetta[2], Chiara Florindi[2,3], Nicol Spallacci[4], Soraia Flammini[4], Mattia Zangoli[4], Francesco Lodola[2,3], Mario Caironi[2], Jaime Martin[5], Francesca Di Maria[4]* and Guglielmo Lanzani[1,2]**

[1]Dipartimento di Fisica, Politecnico di Milano; Piazza L. da Vinci 32, Milan, 20133 Italy.

[2]Center for Nano Science and Technology, Istituto Italiano di Tecnologia; Via Rubattino 81, Milan, 20134 Italy.

[3]Department of Biotechnology and Biosciences, University of Milan-Bicocca, Piazza della Scienza, 2, Milan, 20126 Italy.

[4]Institute for Organic Synthesis and Photoreactivity (ISOF), National Research Council of Italy (CNR); Via P. Gobetti 101, Bologna, 40129 Italy.

[5]Universidade da Coruña, Campus Industrial de Ferrol, CITENI; Esteiro, Ferrol, 15403 Spain.

***Corresponding author:** ludovico.aloisio@liu.se (L. Aloisio), guglielmo.lanzani@iit.it (G. Lanzani), francesca.dimaria@isof.cnr.it (F. Di Maria).

†Current institution (L. Aloisio): Laboratory of Organic Electronics, Department of Science and Technology, Linköping University, SE-60174 Norrköping, Sweden.

The PDF file includes:

Supplementary Text

Figs. S1 to S14

Tables S1 to S2

Supplementary text

Electrical Cell Coupling Model: Numerical Simulations

Transfer characteristics obtained from DTTO OFETs in both standard top gate bottom contact and bottom gate bottom contact architectures indicate that device operation is strongly affected by charge trapping. In both cases, the transfer curves display relatively pronounced hysteresis and a threshold voltage shifted towards negative values, consistent with a high density of traps and slow interfacial or bulk charging processes during the gate sweep. The extracted saturation mobility is modest, in the order of 10^{-6} cm²/Vs for the top gate bottom contact device and further reduced to about 5×10^{-7} cm²/Vs for the bottom gate bottom contact device, suggesting more severe transport limitation in the latter, possibly due to differences in the semiconductor dielectric interface and or effective contact resistance. In both architectures, no n-type operation was measurable.

To understand whether the electrical communication between adjacent cells could be mediated by electronic transport in DTTO fibers, we developed an equivalent electrical circuit model representing a dual patch-clamp configuration (Figure 4D). Each of the two cells is modelled as a membrane circuit composed of a membrane resistance R_m in parallel with a membrane capacitance C_m. The intracellular space of each cell is connected to a bath (extracellular ground) through a resistance R_{bath}, and the pipette access resistance R_s is placed in series with the internal circuit to reflect the pipette-cell interface. The intercellular connection (native gap junction or a DTTO fiber) is represented by a parallel combination of a resistance R_j and a capacitance C_j, accounting respectively for resistive (ionic or electronic) conduction and capacitive (displacement) currents across the junction.

In the steady-state regime (i.e., for long times after the transient response has settled), capacitive effects vanish, and the system behaves as a purely resistive network. We begin by applying Kirchhoff's laws under the assumption that $\frac{dV}{dt} = 0$ for all capacitive elements.

Let V_A and V_B be the intracellular potentials of Cell A and Cell B, respectively. When a constant current I_{inj} is injected into Cell A (e.g. 500 pA), the current divides into two paths:

1. Through the membrane of Cell A and its associated bath resistance:

$$I_{mA} = \frac{V_A}{R_{mA} + R_{bath}}$$

2. Through the junction into Cell B:

$$I_j = \frac{V_A - V_B}{R_j}$$

Thus, Kirchhoff's law at Cell A gives:

$$I_{inj} = \frac{V_A}{R_{mA} + R_{bath}} + I_j = \frac{V_A}{R_{mA} + R_{bath}} + \frac{V_A - V_B}{R_j}$$

At the same time, the current entering Cell B via the junction must be dissipated across its own membrane and bath:

$$\frac{V_B - V_A}{R_j} = \frac{V_B}{R_{mB} + R_{bath}}$$

Solving these equations simultaneously yields the steady-state values of V_A and V_B, which can be used to estimate the effective resistance of the junction. This forms the basis for interpreting experimental current clamp data at long time scales, where only resistive contributions dominate.

To capture the full dynamic response of the system, including both transient and steady-state behaviors, we consider the time-dependence of the intracellular potentials $V_A(t)$ and $V_B(t)$ under current stimulation of Cell A (current injected into Cell A is I_{inj}=500 pA, for 20 ms). This current is distributed among membrane charging, membrane leak current, resistive current through the junction, and capacitive current across the junction. Rearranging the terms, this leads to:

$$C_{mA}\frac{dV_A}{dt} = I_{inj}(t) - \frac{V_A}{R_{mA} + R_{bath}} + \frac{V_B - V_A}{R_j} + C_j\frac{d}{dt}(V_B - V_A)$$

Cell B is not actively stimulated, so it only receives current from Cell A. The incoming current through the junction is both resistive and capacitive. Balancing these currents yields:

$$C_{mB}\frac{dV_B}{dt} = -\frac{V_B}{R_{mB} + R_{bath}} - \frac{V_B - V_A}{R_j} - C_j\frac{d}{dt}(V_B - V_A)$$

We numerically integrated these coupled equations in MATLAB to generate $V_A(t)$and $V_B(t)$traces under the same stimulation protocol used experimentally (500 pA, 20 ms). The simulations were used to compare conditions and to assess the sensitivity of the responses to

junctional parameters. Across the explored physiological range, varying C_j introduced only small changes in the predicted dynamics, which were not clearly discernible experimentally.

Electrical Cell Coupling Model: Electronic Transport

As a rough order of magnitude estimate, we considered the apparent hole mobility extracted from transistors based on one-dimensional DTTO aggregates, $\mu \approx 10^{-5}$cm^2/Vs, as a conservative lower bound for transport along the fibrous state. This value is likely underestimated, as discussed in the main text, but the intrinsic mobility of an individual fiber is not expected to be higher by enough orders of magnitude to qualitatively change the considerations below.

Starting from this lower bound, simple estimate of the resistance expected for an intercellular DTTO junction can be made. The resistivity of a fiber can be expressed as $\rho = 1/n\mu q$, where n is the charge carrier density, μ the carrier mobility, and q the elementary charge. The electrical resistance of a fiber of length L and cross-sectional area A is therefore given by $R = \rho L/A$; hence $R = L/(An\mu q)$. This estimate is necessarily approximate, but it is useful to assess the order of magnitude involved.

DTTO is a wide-gap small-molecule semiconductor and is therefore expected to exhibit a negligibly small intrinsic electronic carrier density. To obtain a realistic upper-bound estimate for n, we considered the possibility of environmentally induced doping, as commonly reported for organic semiconductors, which could increase the carrier density up to 10^{15} cm^{-3}. Accordingly, in one fiber, the product $n \times \mu$ could range between 10^9 and 10^{10} (cmVs)$^{-1}$. For a 10 nm segment representing the junction, the corresponding electrical resistance would therefore be 10^2-10^3 GΩ. This is a very large value compared with the junction resistance obtained from the numerical solution of the equivalent electrical circuit and can hardly justify the observed functionality on the basis of intrinsic electronic transport alone.

Electrical Cell Coupling Model: Current Relaxation Dynamics

As shown in fig. S12, under humid conditions and constant applied bias, the current did not remain constant over time, but instead decayed, indicating that the response is not purely ohmic. When the applied voltage was returned to 0, a smaller current of opposite sign was observed before the signal gradually relaxed back toward baseline. This behavior suggests that the response includes a time dependent component with partial retention of the polarized state established under bias. Consistent with the current voltage measurements, these observations

further support the view that the electrical behavior of hydrated DTTO aggregates cannot be described as simple electronic transport alone.

A different behavior was observed for H_2O alone. In this case, the absolute current amplitude should not be directly compared with that of DTTO containing samples, because the amount of material effectively present between the electrodes was different. The relaxation dynamics, however, provide a clear qualitative comparison. H_2O showed a much slower decay than the DTTO containing sample, indicating that the response observed in the presence of DTTO cannot be attributed simply to ionic leakage through absorbed water. Rather, the fiber network appears to modify the hydrated electrical response, promoting a distinct relaxation behavior under bias. The larger long time residual fraction observed for H_2O alone, about 10% of the initial current, further supports that the process remains different from that observed in DTTO, where the residual fraction stayed substantially lower even at 90% RH.

Taken together, these results show that hydration is essential, but that the DTTO fiber network reshapes both the relaxation dynamics and the persistent current fraction.

Electrical Cell Coupling Model: Electrochemical Impedance Spectroscopy

Electrochemical impedance spectroscopy was used to further analyze the humidity dependent electrical response in the frequency domain (fig. S13). The spectra of DTTO at 90% RH, H_2O alone, and DTTO + H_2O were all well reproduced using the same equivalent circuit framework, consisting of two parallel ($R \parallel CPE$) branches connected in series (fig. S14). In this model, the constant phase element accounts for nonideal capacitive behavior, while in the limiting case $N = 1$ it becomes an ideal capacitor, so that the corresponding branch reduces to a standard parallel RC element.

The fit results, reported in Table S2, further support the qualitative differences already evident in the spectra. H_2O alone could be described by two ideal *RC* type contributions, consistent with a predominantly ionic blocking response without a pronounced dispersive component. By contrast, DTTO at 90% RH required an additional nonideal contribution, indicating the presence of a distributed and dispersive electrical response not present in H_2O. This difference is also reflected in the resistance values. DTTO at 90% RH showed substantially larger resistances, with R_1 increasing from 21.3 MΩ for H_2O to 223.7 MΩ for DTTO and R_2 increasing from 59.1 kΩ to 4.95 MΩ, corresponding to roughly one and nearly two orders of magnitude, respectively.

The mixed DTTO + H_2O sample showed intermediate resistance values, with R_1 = 30.08 MΩ and R_2 = 225.1 kΩ, consistent with the additional conductive contribution of water. However, its response could not be reduced to that of H_2O alone, since the fit still required a dispersive contribution associated with the presence of DTTO. Thus, although water lowers the effective resistance of the system, the fiber network still modifies the electrical response in a way that remains distinct from simple hydrated ionic behavior.

Taken together, these fits show that the presence of DTTO does not simply add a conductive path in parallel with H_2O but introduces an additional distributed and dispersive contribution that reshapes the hydrated electrical response.

Supplementary Figures

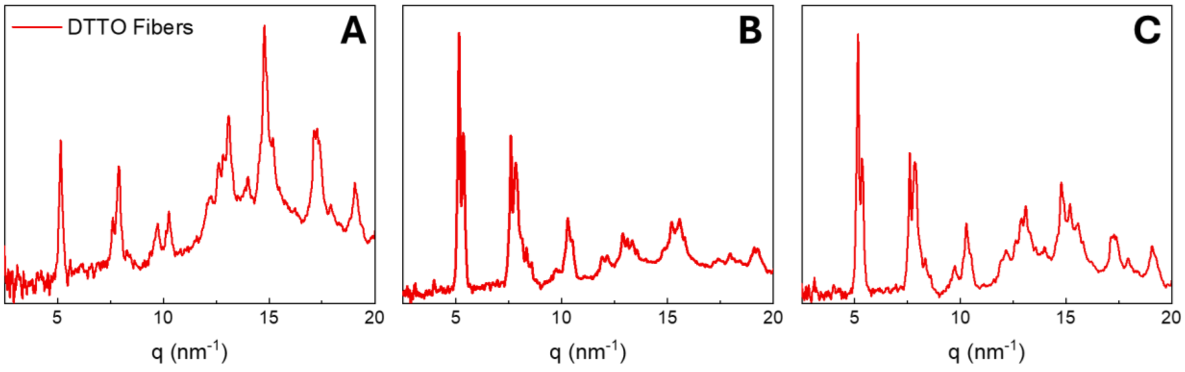

Fig. S1.

*2D GIWAXS profiles for DTTO fibers integrated (**A**) in plane, (**B**) out of plane and (**C**) integrated over the whole area.*

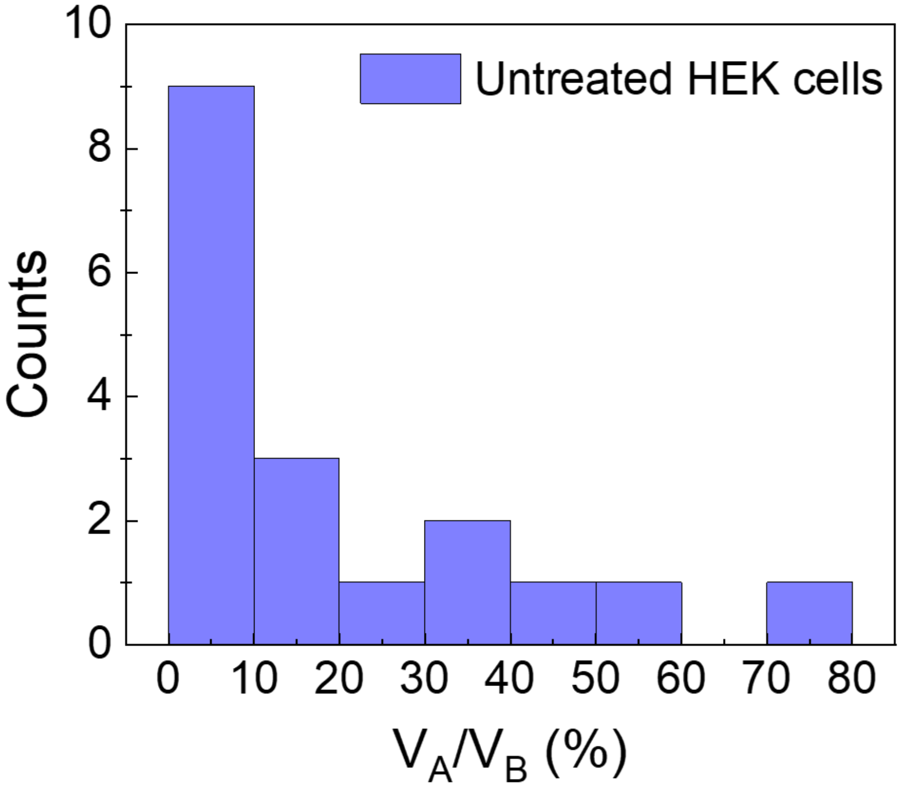

Fig. S2.

Distribution of signal propagation between adjacent untreated HEK 293T cells measured by double patch clamp. Signal propagation is expressed as the membrane depolarization recorded in the second cell relative to that of the stimulated first cell. 9 out of 18 cell pairs showed little or no detectable communication, with signal propagation below 10%, whereas the remaining pairs exhibited appreciable intercellular coupling. This heterogeneity is consistent with the occasional formation of functional endogenous gap junctions in HEK 293T cells.

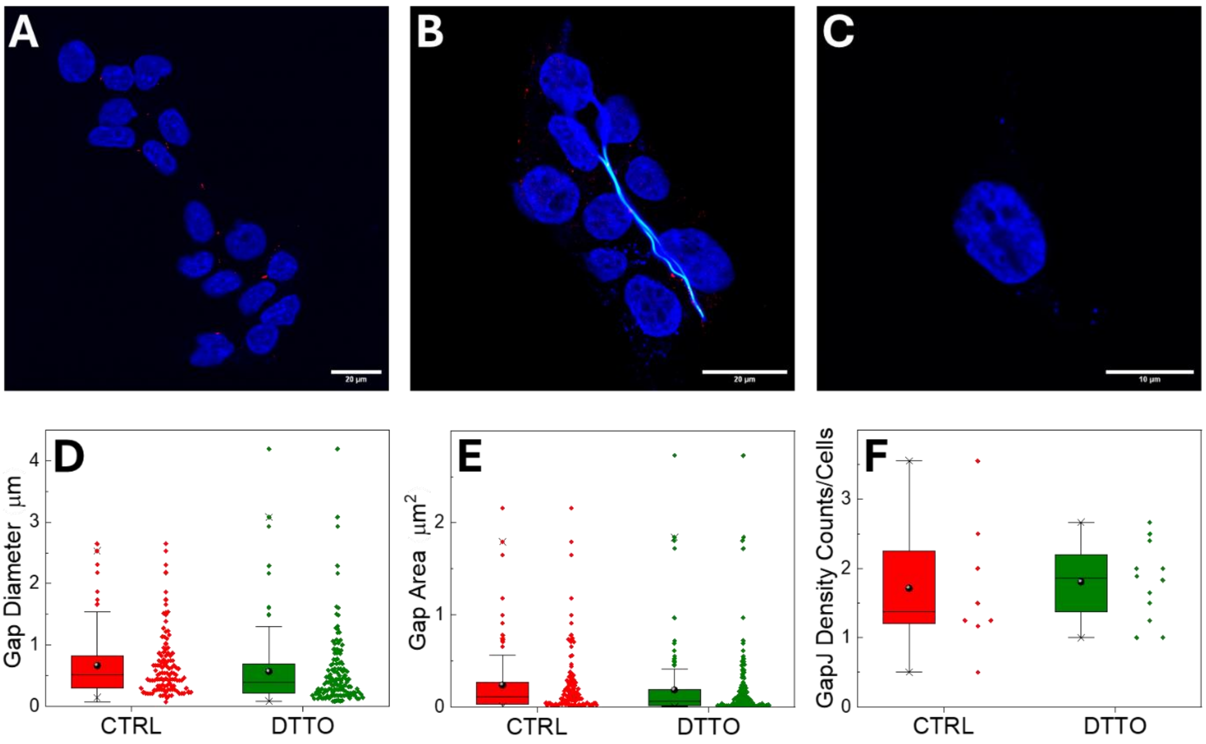

Fig. S3.

*(**A**-**C**) Immunostaining confocal images of HEK and Connexin 43 (Blue – DAPI, Green – DTTO, Red – Connexin 43). Representative images of Cell without DTTO (**A**) with DTTO (**B**) and Single cells not expressing Connexin (**C**). (**D**-**F**) Data representing the cluster of Connexing43 dimension (**D** and **E**) and density (**F**), calculated as number of connexin cluster divided by number of cells. No significative differences are visible between control and DTTO-treated Cells. (N_{CTRL}=8 Samples, $N_{gap\text{-}CTRL}$=114 gap, N_{DTTO}=14, Samples, $N_{gap\text{-}DTTO}$=139).*

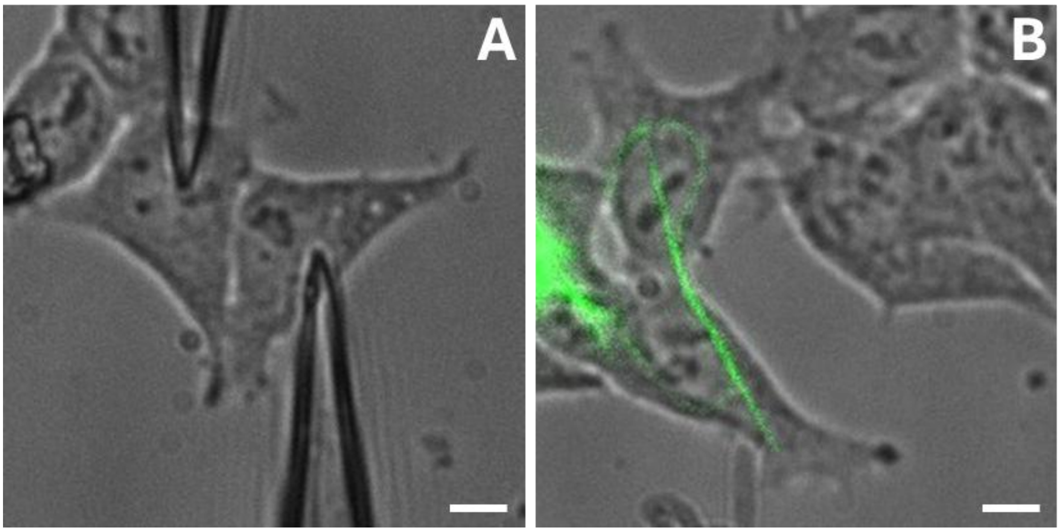

Fig. S4.

*40X magnification images of HEK-293T cells couples before patch clamp experiment, clearly showing a membrane separating them: (**A**) control sample and (**B**) fiber sample. Scale bars: 5 µm.*

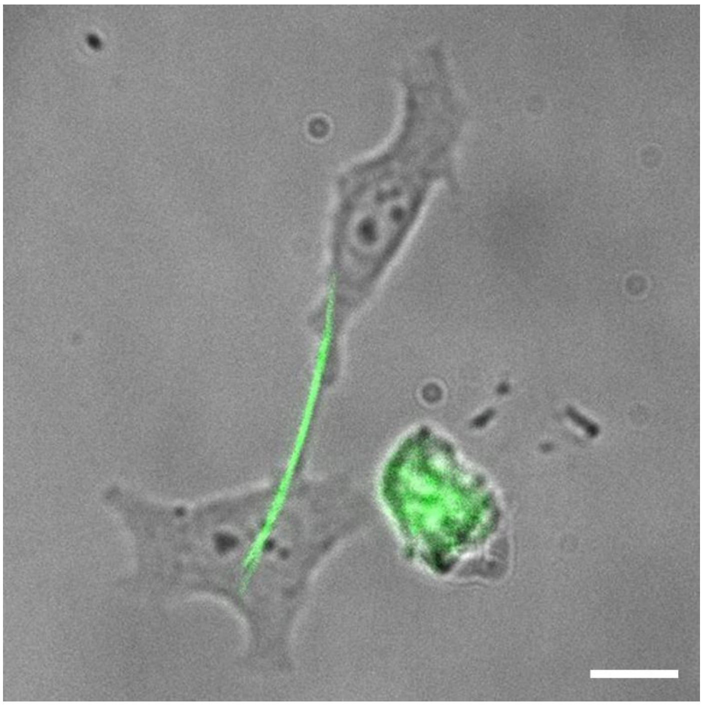

Fig. S5.

40X magnification image of HEK-293T cells with completely separated membranes, connected by a DTTO fiber. Scale bar: 5 µm.

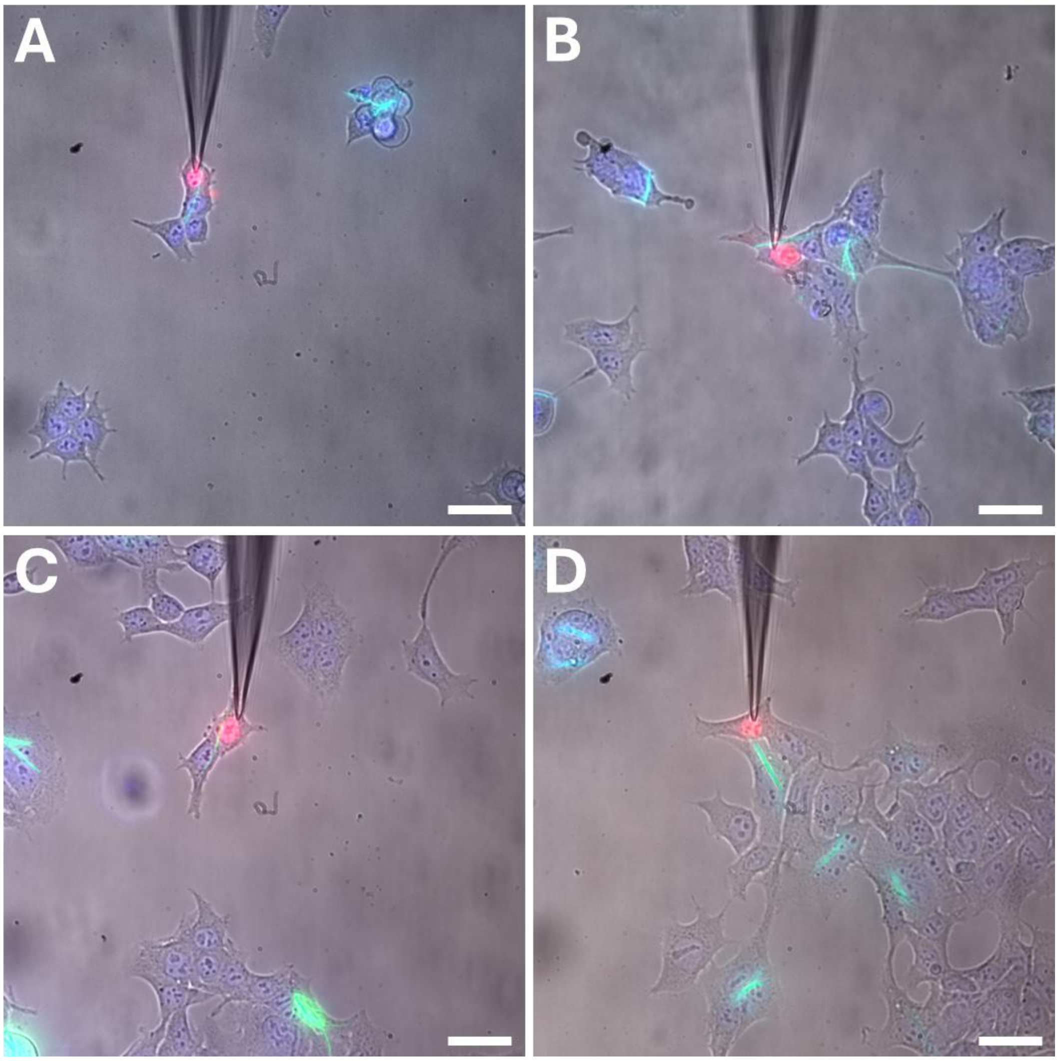

Fig. S6.

*(**A**-**D**) Additional 40X magnification images of HEK-293T cells, connected by DTTO fibers, and patched in whole-cell configuration with a capillary loaded with propidium iodide to verify the presence of pores between cells. All figures are superimposition of brightfield view with images collected through different epifluorescence channels: blue (epifluorescence: excitation 350/50 nm, dichroic 400 nm long pass, emission 460/50 nm) showing Hoechst and DTTO fibers; green (epifluorescence excitation 470/40 nm, dichroic 495 nm long pass, emission 525/50 nm) showing DTTO fibers; red (epifluorescence excitation 540/25 nm, dichroic 565 nm long pass, emission 605/55 nm) showing PI. Scale bars: 25 µm.*

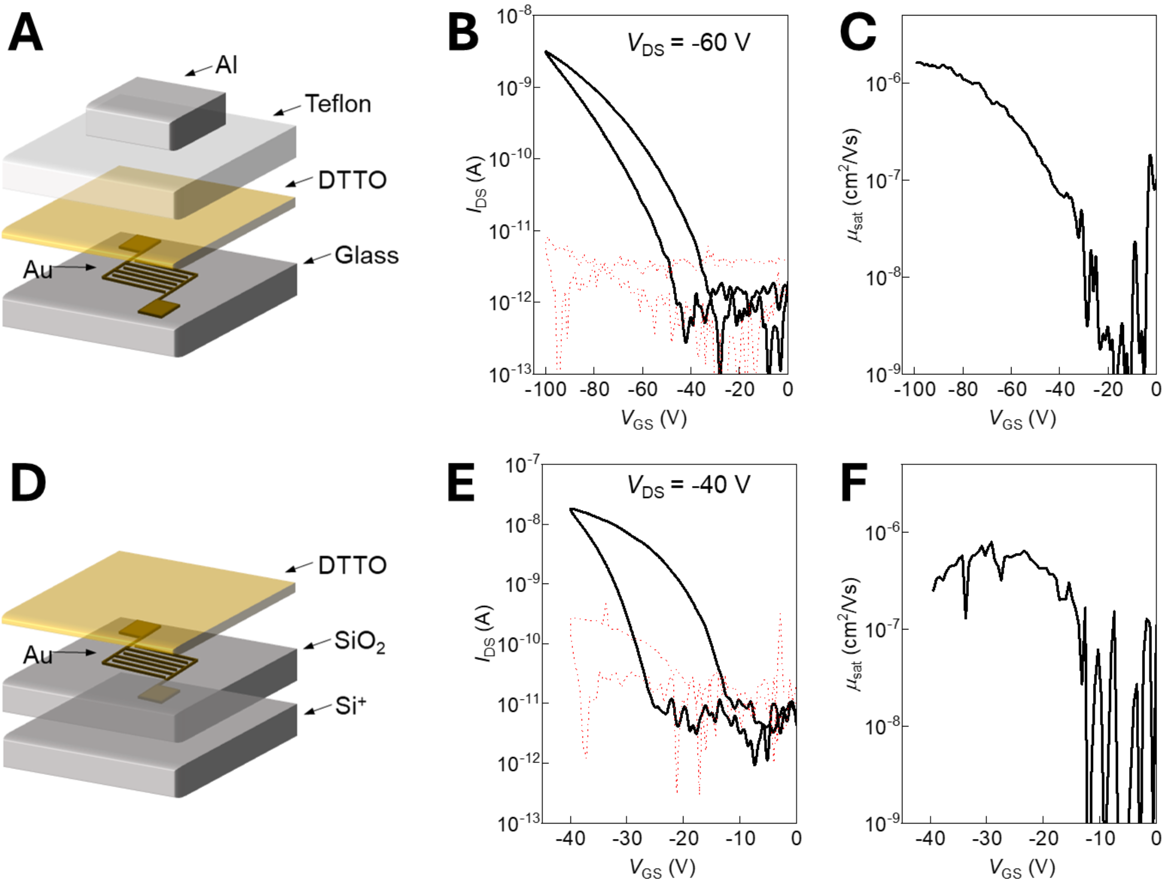

Fig. S7.

*(**A**) Schematic of a DTTO OFET in the top gate bottom contact architecture. (**B**) Representative transfer characteristics measured for the top gate device. (**C**) Field effect mobility extracted in the saturation regime from the transfer data. (**D**) Schematic of a DTTO OFET in the bottom gate bottom contact architecture. (**E**) Representative transfer characteristics measured for the bottom gate device. (**F**) Field effect mobility extracted in the saturation regime from the transfer data.*

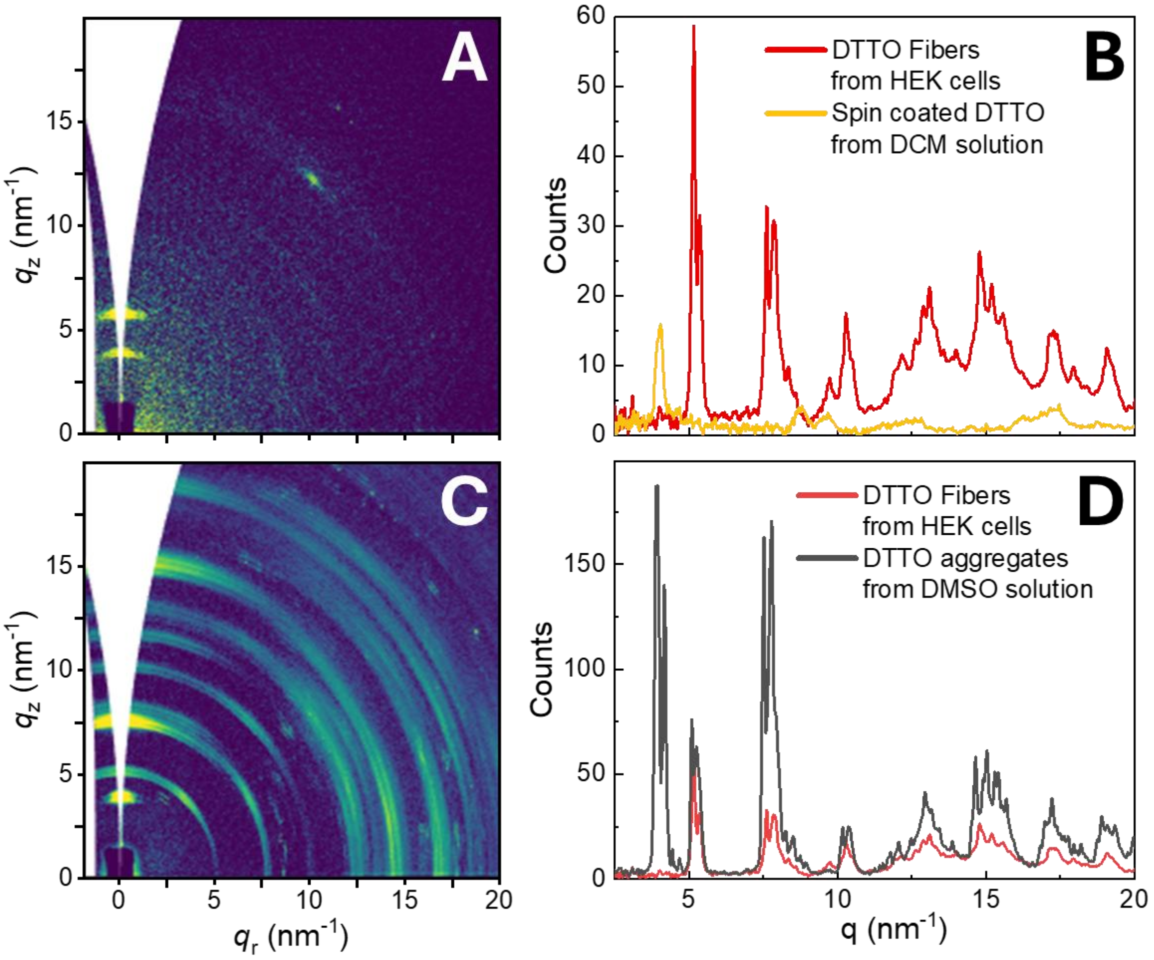

Fig. S8.

*(A) 2D GIWAXS pattern and (**B**) corresponding integrated GIWAXS profile of a DTTO thin film prepared by spin coating from a dichloromethane solution at 1000 rpm for 60 s, followed by annealing at 100 °C. (**C**) 2D GIWAXS pattern and (**D**) corresponding integrated GIWAXS profile of DTTO aggregates prepared by evaporation from a DMSO solution. All profiles were obtained by integration over the whole 2D pattern. The red traces in B and D correspond to the integrated GIWAXS profile of DTTO fibers formed in HEK 293T cells and subsequently extracted, included as a reference for comparison.*

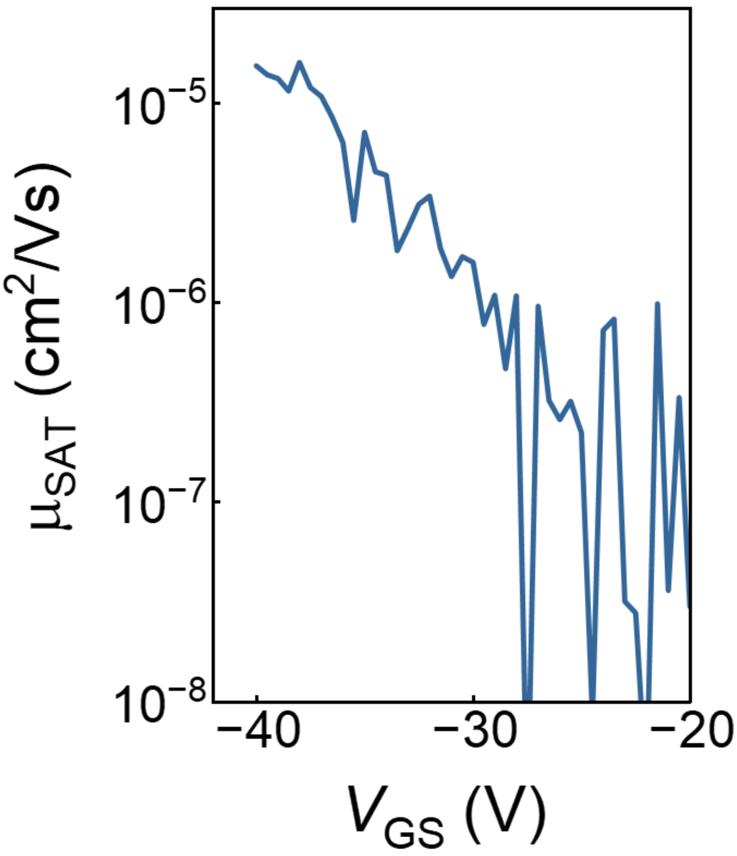

Fig. S9.

Field effect mobility for DTTO aggregates formed by DMSO solution evaporation, extracted in the saturation regime from the transfer data reported in Figure 4A.

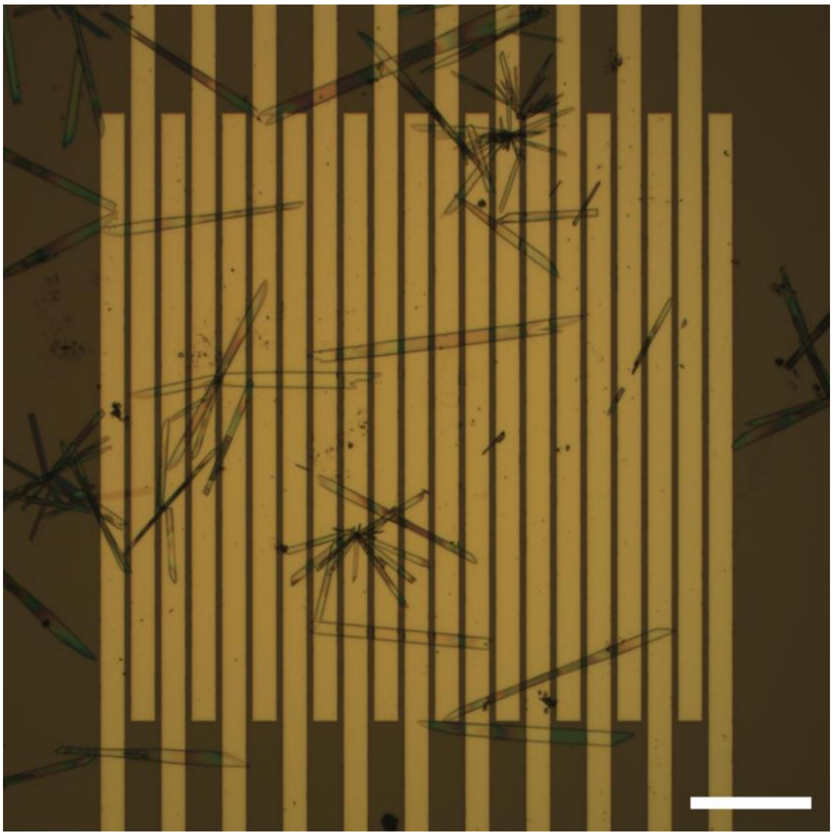

Fig. S10.

Reflection optical image of DTTO aggregates, grown by DMSO solution evaporation, deposited on gold interdigitated electrodes used for electrical measurements. Scale bar: 50 µm.

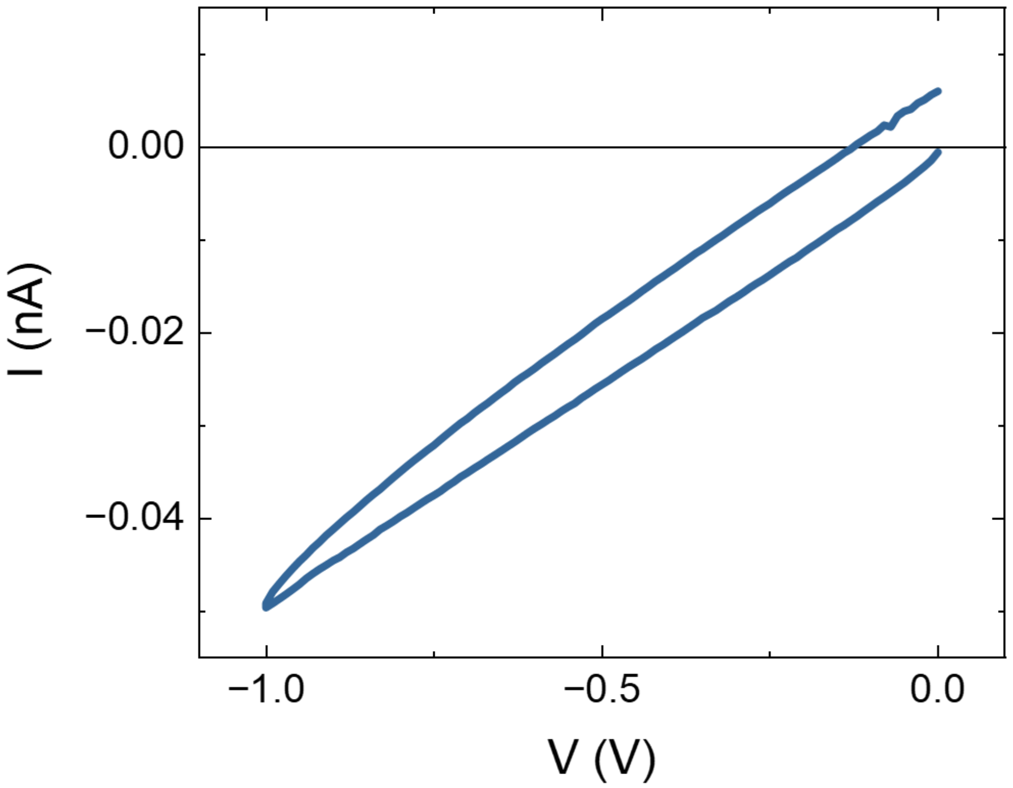

Fig. S11.

I-V characteristics of the base substrate measured at 90% relative humidity. The substrate is the same as the one used for DTTO aggregates measurements.

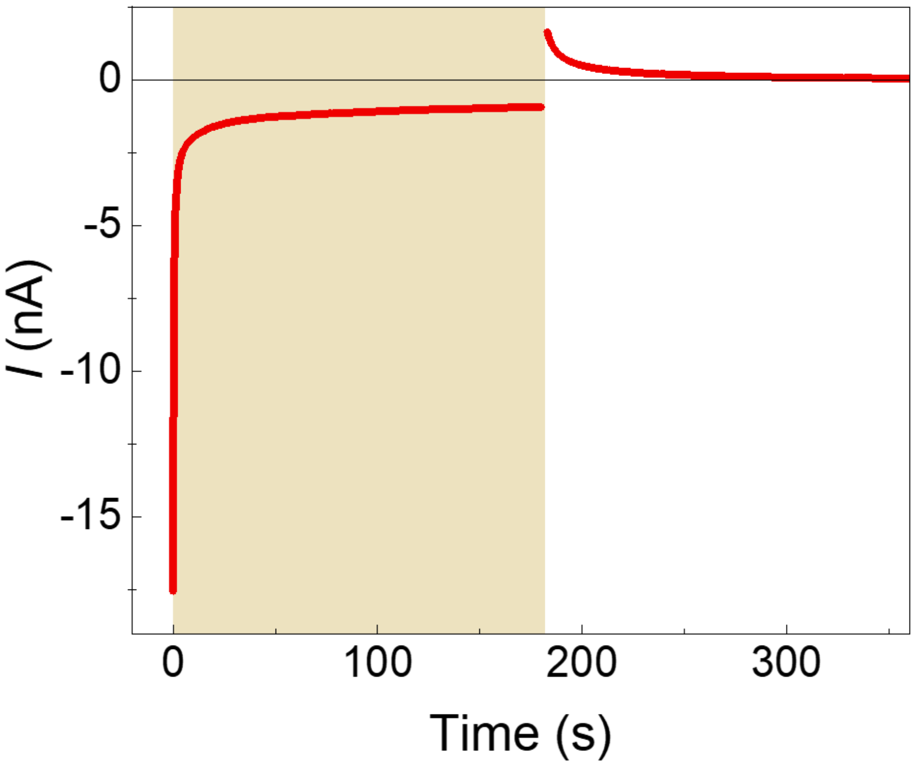

Fig. S12.

Time dependent current measured on DTTO aggregates at RH 90% during application of a constant bias of −1 V for 180 s, highlighted by the shaded region, followed by continued measurement after ΔV was returned to 0.

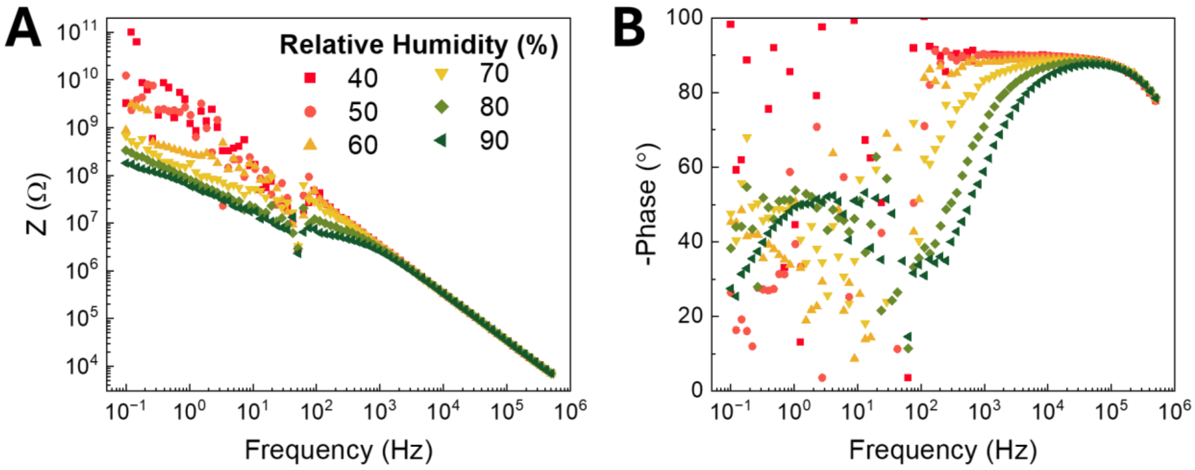

Fig. S13.

*Electrochemical impedance spectroscopy of DTTO aggregates, grown by DMSO solution evaporation, at different relative humidity values. Bode plots showing (**A**) impedance modulus and (**B**) phase angle as a function of frequency.*

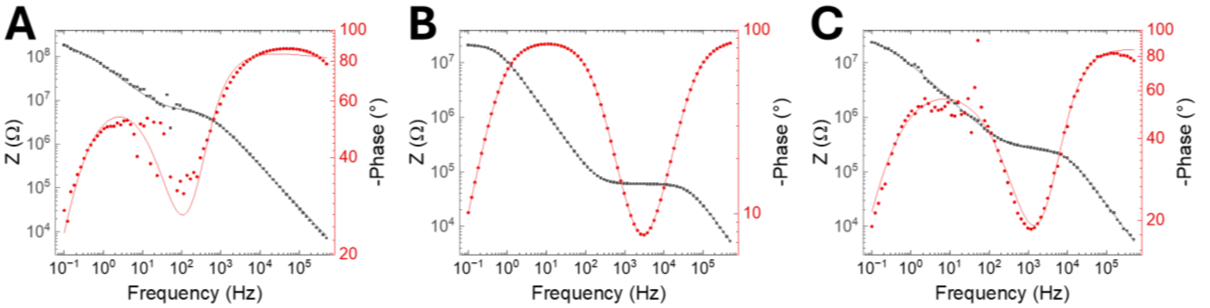

Fig. S14.

*Electrochemical impedance spectroscopy of (**A**) DTTO aggregates at 90% relative humidity, (**B**) H_2O, and (**C**) DTTO aggregates + H_2O, shown as a Bode plot of the impedance modulus (gray) and phase angle (red). Experimental data are shown as scatter points, and the fitted curves as solid lines.*

Supplementary Tables

Parameter	Value	Description
R_{pA}	3×10^{6} Ω	Pipette resistance Cell A
R_{pB}	3×10^{6} Ω	Pipette resistance Cell B
R_{mA}	1×10^{8} Ω	Membrane resistance Cell A
R_{mB}	1×10^{8} Ω	Membrane resistance Cell B
R_{bath}	1×10^{3} Ω	Bath resistance
R_{j}	1.27×10^{8} Ω (Fiber)<br>1.25×10^{9} Ω (CBX)	Gap junction resistance
C_{mA}	10×10^{-12} F	Membrane capacitance Cell A
C_{mB}	10×10^{-12} F	Membrane capacitance Cell B
C_{j}	From 10×10^{-13} F to 10×10^{-15} F	Gap junction capacitance

Table S1.

Parameters used to replicate experimental data in the MATLAB code used to solve the equivalent electrical circuit.

**Sample**	**R_1**	**Q_1**	**N_1**	**R_2**	**Q_2**	**N_2**
**DTTO RH90%**	223.7 MΩ	3.71 nS·s^N (C=2.71nF)	0.761	4.95 MΩ	61.3 pS·s^N (C=61.3 pF)	1
**H_2O**	21.3 MΩ	13.4 nS·s^N (C=13.4 nF)	1	59.1 kΩ	60.4 pS·s^N (C=60.4 pF)	1
**DTTO + H_2O**	30.08 MΩ	24.9 nS·s^N (C=9.96 nF)	0.715	225.1 kΩ	71.3 pS·s^N (C=71.3 pF)	1

Table S2.

Fitting parameters from electrochemical impedance spectroscopy data using an equivalent circuit consisting of two (R || CPE) elements connected in series. Q is the constant phase element pre-factor and N its exponent. When N = 1, the constant phase element becomes an ideal capacitor, so that the corresponding branch reduces to a standard R || C element. The corresponding capacitance value is reported in parentheses.
